# Digitale Pflegebetten in der Langzeitpflege – Unterstützung, Entlastung für Pflegende oder nur Illusion? Eine ethnografisch-explorative Studie

**DOI:** 10.1007/s00391-023-02267-z

**Published:** 2024-01-02

**Authors:** Sven-Nelson Ruppert, Martina Hasseler

**Affiliations:** Fakultät Gesundheitswesen, Ostfalia Hochschule für angewandte Wissenschaften – Hochschule Braunschweig/Wolfenbüttel, Robert-Koch-Platz 8a, 38440 Wolfsburg, Deutschland

**Keywords:** Digitale Pflege, Digitale Assistenzsysteme, Pflegeprozess, Altenpflege, Deutschland, Digital care, Digital assistance systems, Care process, Nursing care, Germany

## Abstract

**Hintergrund:**

Die Digitalisierung von Pflegedingen, hier dem Pflegebett, ist eine mögliche Konsequenz auf den allgemeinen Personal- und Ressourcenmangel in der beruflichen Pflege und eine Zunahme pflegebedürftiger, hochaltriger Menschen in der Langzeitpflege. Durch in das Pflegebett integrierte Sensoren und Schnittstellen zur Datenübertragung bzw. Anschluss an ein Monitoring sollen für beruflich Pflegende Unterstützungs- und Entlastungsmomente generiert werden sowie das pflegerische Outcome gesteigert werden. Eine pflegewissenschaftliche Begleitforschung untersucht die Implementierung von 97 digitalen Pflegebetten in einer Langzeitpflegeeinrichtung der Altenhilfe.

**Methode:**

Mixed-Methods-Forschungsansatz über 24 Monate.

**Ergebnis:**

Das Fehlen bzw. Umsetzen eines konsequenten Pflegeprozesses, ungünstige Begleitumstände, interdependente Kontextfaktoren und ein unzureichendes Implementierungsmanagement wirken sich nachteilig auf die Inbetriebnahmen und Integration der digitalen Pflegebetten in die Versorgungsroutinen aus.

**Schlussfolgerung:**

Für eine erfolgreiche Implementierung digitaler Assistenzsysteme (dAS) müssen diese neben einer zuverlässigen technischen Anbindung im Pflegeprozess berücksichtigt werden. Im Weiteren erforderlich sind ein Implementierungsmanagement sowie die Überprüfung und Anpassung aller relevanten Kontextfaktoren.

**Zusatzmaterial online:**

Zusätzliche Informationen sind in der Online-Version dieses Artikels (10.1007/s00391-023-02267-z) enthalten.

## Abstract (en)

**Background:**

The digitalization of care items, in this case the care bed, is a logical consequence of the general shortage of personnel and resources in professional care and an increase in the number of very old people in need of long-term care. Sensors integrated into the nursing bed, interfaces for data transmission and connection to a monitoring system are generated for moments of support and relief for professional nurses and to increase the nursing outcome. Accompanying nursing research examines the implementation of 97 digital care beds in a long-term care facility for old people.

**Method:**

Mixed methods research approach over 24 months

**Results:**

The lack or implementation of a consistent care process, unfavorable accompanying circumstances, interdependent contextual factors and an insufficient implementation management have a negative impact on the implementation and integration of the digital care beds into the care routines.

**Conclusion:**

For a successful implementation of digital assistance systems (dAS), they have to be considered in the care process besides a reliable technical connection. Furthermore, an implementation management as well as the review and adaptation of all relevant context factors are required.

## Hintergrund und Fragestellung

Das Statistische Bundesamt hat auf Grundlage der 15. koordinierten Bevölkerungsvorausberechnung eine Zunahme der Pflegebedürftigkeit in Deutschland berechnet. Für 2035 zeigt die moderate Prognose 5,6 Mio. und bei steigender Pflegequote bis zu 6,3 Mio. pflegebedürftige Personen [1]. Für die Langzeitpflege bedeutet dies, dass es bis 2060 zu einer Verdopplung der Pflegebedürftigen kommt [2]. Bei den Hochaltrigen über 80 Jahre wird ein dynamischer Zuwachs erwartet [3]. Parallel dazu wird bis 2035 ein Personaldefizit beruflich Pflegender von 307.000 prognostiziert [4]. Rund 100.000 Stellen für Pflegende werden in der Langzeitpflege fehlen [5]. Diskutiert wird, ob eine Digitalisierung den Personalmangel in der Langzeitpflege kompensieren und weiter die Versorgung der Bewohner gewährleisten kann [6]. Ein Ansatz wäre, den zentralen Gegenstand in der Pflege, das Pflegebett, zu digitalisieren. Durch Sensoren und Monitoring können frühzeitig Versorgungsrisiken z. B. Gewichtsverlust und Immobilität erkannt werden. Pflegefachpersonen reagieren proaktiv auf die Risikoeinschätzung der Pflegebetten durch Anpassung des Pflegeprozesses und der Pflegemaßnahmen.

In einer neu eröffneten Langzeitpflegeeinrichtung der Altenhilfe in Niedersachsen, ohne Versorgungsschwerpunkt, mit 5 Wohnbereichen und 97 Pflegeplätzen wurden gleich viele digitale Pflegebetten (im Weiteren nur Pflegebett) neu angeschafft. Die intendierten Ziele durch die Anschaffung sind, die Pflegefachpersonen und Pflegehelfer*innen zu entlasten und die Versorgungsqualität, bezogen auf Sturz, Dekubitus und ungewollten Gewichtsverlust, zu verbessern. Der Querschnitt der Bewohnenden benötigt wenig pflegerische Unterstützung und ist überwiegend mobil. Aufgrund des Fachkräftemangels konnten während der Forschung nicht alle Planstellen für Pflegende besetzt werden. In die Pflegebetten sind eine Waage und Drucksensoren integriert. Diese registrieren z. B. im Rahmen der Bed-Exit-Funktion (BEF), ob eine Person das Bett verlassen will, verlassen hat oder sich wieder hinlegt. Über Schnittstellen können Informationen an ein Monitoring übermittelt sowie Risiko- oder Alarmmeldungen ausgegeben werden. Zudem sind ein Datentransfer an eine Pflegesoftware und eine automatische Dokumentation möglich.

Die drittmittelgeförderte pflegewissenschaftliche Begleitforschung zur Implementierung der Pflegebetten untersuchte, ohne Einflussnahme auf den Implementierungsprozess, ob die intendierten Ziele erreicht wurden. Dieser Beitrag widmet sich einer Frage, ob es durch die Implementierung der Pflegebetten zu einer Veränderung der pflegerischen Versorgung kommt. Kriterien dafür sind Pflegearbeit, Pflegeprozesses, Digitalisierung und Technikanwendung. Ein zusätzlicher Fokus liegt auf dem Implementierungsprozess und den assoziierten Kontextfaktoren, beispielsweise Personalmangel.

## Studiendesign

Der Implementierungsprozess der Pflegebetten stellt aufgrund der interdependenten Komponenten wie der Herstellung einer digitalen Infrastruktur, mit Schnittstellen zu Pflegesoftware und -dokumentation, Mitarbeiterschulungen, Überarbeitung von Pflegeroutinen, Veränderung der Organisations- und Handlungsstrukturen sowie den pflegerischen Verantwortungsstrukturen u. v. m., eine komplexe Intervention dar [7]. Der Mixed-Methods-Forschungsansatz berücksichtigt diese Komplexität. Eine quantitative Analyse der Inzidenzen der genannten Qualitätsindikatoren vor und nach Implementierung der Pflegebetten entfiel, da keine Konnektivität zur Pflegesoftware und somit kein Monitoring möglich waren. Zudem lieferten die auch offline nutzbaren Bettenwaagen keine reliablen Werte, im Vergleich zu den Referenzwerten der Sitzwaage. Technikaffinität gilt als Prädiktor für ein erfolgreiches Implementierungsmanagement [8]. Die verwendete ATI-Skala misst die Technikaffinität bei den Pflegenden (Pflegefachpersonen und -helfer*innen des Stammpersonals) [9]. Sie enthält 9 Items. Eine 6fach skalierte Likert-Skala erlaubt Antworten von „stimmt gar nicht“ bis „stimmt völlig“. Die ATI-Skala gilt als ein reliables und konstruktvalides Instrument [10]. Mittels eines entworfenen Fragebogens beurteilten die Pflegenden den Implementierungsprozess. Die qualitative Forschung folgt dem Paradigma der Ethnografie. Diese methodenplurale Forschung ermöglicht, in unbekannten Gruppen oder sozialen Einheiten deren Handlungsweisen und Wissensformen durch eine systematische teilnehmende Beobachtung sowie durch formelle wie informelle Gespräche, Daten zu erheben und die räumlich-dingliche Konstellation des Feldes zu analysieren [11]. Alle Forschungsdaten werden durch den Autor erhoben. Teilnahmevoraussetzungen sind Freiwilligkeit, die Zugehörigkeit zum pflegerischen Stammpersonal sowie eine schriftliche Zustimmung zu Datenerhebung und -auswertung.

Der 24-monatige Forschungsprozess eruiert, ob die Implementierung der Pflegebetten zu Veränderungen in der Pflegearbeit, im Pflegeprozess geführt haben. Es wurden 3 mehrtägige Beobachtungs- und Interviewphasen durchgeführt.

Anfänglich waren die digitalen Komponenten deaktiviert, die Pflegebetten offline. Es wurde ein Gesamteindruck von der Pflegeeinrichtung und den pflegerischen Handlungsroutinen erhoben. Erste technische Probleme in der zweiten Beobachtungsphase verhinderten eine Verbindung mit der Pflegesoftware. Beobachtet wurde, wie die Bettenwaagen und die BEF von den Pflegenden eingesetzt wurden, und ob der Pflegeprozess zur Anwendung kommt. In der dritten Phase, bei anhaltenden Problemen, wurde die Überführung der nutzbaren Optionen in die Versorgungsroutinen, den Pflegeprozess, weiterbeobachtet, und wie die Pflegenden die technischen Defizite mit den vorhandenen Offline-Möglichkeiten der Pflegebetten kompensierten, ihre Pflegehandlungen adaptierten.

Mit 6 langjährig berufserfahrenen Pflegefachpersonen sowie 4 jungen Pflegehelfer*innen mit weniger als 5 Jahren Berufserfahrung wurden während der Arbeitszeit leitfadengestützte Interviews (Zusatzmaterial online: Interviewleitfaden), episodische und später ero-epische Gespräche geführt. Vorteil dieser Art der mündlichen Befragung ist, dass die interviewten Personen aufgrund der Kombination aus Leitfadenfragen und Erzählungen von subjektivem Wissen und Erfahrungen [12] kontextbezogene Erfahrungen beisteuern [11]. Initial wurden die Einrichtungsleitung und Pflegedienstleitung interviewt, abschließend nur die Einrichtungsleitung. Angesprochen wurden die Intention zur Beschaffung der Pflegebetten, der Implementierungsprozess und im zweiten Interview, welche Erfahrungen mit den Pflegebetten gemacht wurden, und wie den Implementierungsproblemen begegnet werden soll. Die Leitfragen für die Pflegenden fokussieren Pflegeroutinen, Aufgabenverteilung und Umsetzung des Pflegeprozesses. Als induktive Ergebnisse der Beobachtungen interessieren, welches Handlungswissen durch Schulungen vermittelt wurde, ob Pflegende dieses anwenden können und im Pflegeprozess Anwendung findet sowie die Anwendungsfreundlichkeit der Pflegebetten. Die Kontextfaktoren Implementierungsmanagement, Schulungen und Personalsituation sind Teil der Datenerhebung.

Die Transkription der 30- bis 60-minütigen Interviews und Gespräche wurde regelgeleitet nach Kuckartz durchgeführt [13]. Die Datenauswertung erfolgte nach der Methode der inhaltlich strukturierenden qualitativen Inhaltsanalyse (Zusatzmaterial online: Kodierungsleitfaden; [13]).

## Ergebnisse

Mit 4,4 von 6,0 Punkten fällt die Technikaffinität bei den Pflegenden gut aus. Der Implementierungsprozess ist mit 4,2 von 6,0 von den berufserfahrenden Pflegefachpersonen etwas schlechter bewertet. Die qualitativen Analyseergebnisse wurden Kategorien zugeordnet. In der Kategorie *Pflege* wurden die Ergebnisse der pflegerischen Versorgung und des Pflegeprozesses beschrieben. Die zweite Kategorie *Digitalisierung/Technikanwendung* beinhaltet Ergebnisse zur IT-Infrastruktur und dem neuen Aspekt der Anwendungsfreundlichkeit der Pflegebetten. Der dritten Kategorie *Kontextfaktoren* sind die Ergebnisse Personalsituation und Implementierungsstrategie zugeordnet.

### Pflege

Die Analyse der Pflegearbeit zeigt zwei Auffälligkeiten. Erstens führt die Fragmentierung der Pflegearbeit dazu, dass Pflegefachpersonen kaum pflegeassoziierten Kontakt mit den Bewohnenden haben, weil überwiegend pflegerische Handlungen von Pflegehelfer*innen durchgeführt werden, bei nicht klar abgegrenzten Zuständigkeiten gegenüber den Vorbehaltsaufgaben der Pflegefachpersonen nach dem Pflegeberufegesetz. *Wir Sachen machen, die wir gar nicht machen dürfen (PHs: 00:47:34)*. Folge ist, dass pflegerische Versorgungsdefizite unzureichend erkannt werden. Zweitens, ein regelkonformer Pflegeprozess wurde nicht beobachtet. Auf die Frage, ob an einen Pflegeprozess überhaupt gedacht wird, antwortet eine berufserfahrene Pflegefachperson: *Nö! (PFP, 00:46:33).*

Die hochgradige Fragmentierung der Pflegearbeit zeigt sich daran, dass Informationssammlung, Ausarbeiten und Anpassen von Pflegemaßnahmen von verschiedenen, unterschiedlich qualifizierten Pflegepersonen durchführt wurden. Eine für die Dokumentation verantwortliche Person schildert, dass aufgrund der hohen Arbeitsbelastung der Pflegenden die systematische Informationssammlung (SIS) nicht fristgerecht erfolgt und Pflegende einen Zettel mit Informationen unter die Bürotür durchschieben, damit ein Maßnahmenplan geschrieben wird. Gleichsam fragmentiert sind bspw. Pflegehandlungen im Kontext des Körpergewichtes. Pflegehelfer*innen wiegen und notieren handschriftlich das Gewicht; Dokumentationsbeauftrage analysieren den Verlauf und passen, wenn nötig, Pflegemaßnahmen an. Die für den Pflegeprozess verantwortlichen Pflegefachpersonen werden kaum beteiligt. Folge ist, dass die Pflegebetten durch Pflegefachpersonen weder nutzenstiftend eingesetzt noch in die Versorgungsroutinen integriert werden können.

### Digitalisierung und Technikanwendung

Die IT-Infrastruktur wird im Ganzen als unzuverlässig beschrieben, v. a. das für Anbindung der Pflegebetten notwendige WLAN. *Die IT ist nicht zuverlässig genug. Toll ist, wenn der PC morgens mal hochfährt, E‑Mails verschickt werden können, (…) da wollen wir gar nicht drüber reden über WLAN (Q: 00:03:19).*

Die Anwendungsfreundlichkeit der Pflegebetten wird von den Pflegenden als kontraproduktiv beschrieben. Die Aktivierung der BEF als auch die Handhabung der Bettenwaage sind zu komplex. (…) *bis wir die eingestellt haben und dieses Komplexe, mit so vielen Knöpfen, das machen sie nicht (L: 00:14:29)*. Und Stromunterbrechungen z. B. bei Reinigungsarbeiten führen zu einem Verlust der im Bett gespeicherten Daten. *Passiert es dann, dass irgendwer den Stecker zieht, (…) sind alle Einstellungen weg (O: 00:12:22).*

### Kontextfaktoren

Die Implementierung der Pflegebetten wird von Kontextfaktoren, beispielsweise der Implementierungsstrategie, Schulungen und Personalmangel, beeinflusst. Unisono berichten die Pflegenden, dass die Idee der Pflegebetten gut ist. Kritisch merken sie an, dass eine Implementierungsstrategie und Schulungen zum Umgang mit den Pflegebetten fehlen. *Es wird einfach immer in irgendetwas hineingestoßen, macht mal, macht mal, macht mal, macht mal, ihr bekommt das alles schon irgendwie hin (L: 00:33:38)*. Die Befragten berichteten, dass nach 24-monatigen Betrieb nur wenige Pflegefachpersonen geschult wurden. *Welche Schulungen, durch wen? (Q: 00:12:36).*

Pflegefachpersonen und Einrichtungsmanagement beschreiben einen ständigen Fachmangel. Die Substitution durch Pflegende aus der Arbeitnehmendenüberlassung (ANÜ) führt zu keiner Entlastung beim Stammpersonal, da die Pflegenden aus der ANÜ nicht in die Pflegebetten eingewiesen wurden.

## Diskussion

Die Ergebnisse zeigen, dass die Implementierung der Pflegebetten nicht die intendierte Entlastung, Unterstützung der Pflegenden und eine Verbesserung der pflegerischen Versorgungsqualität ermöglicht hat. *Ja, ist zwar irgendwo schade, aber ich denke, man muss es dann auch klar so sagen, weil sonst würde man auch nicht bemerken, dass die Betten wie ganz normale Pflegebetten genutzt werden (O: 00:31:25-2)*. Ursächlich sind keine isolierten Probleme, sondern interdependente Faktoren, die sich über Kategoriengrenzen hinweg gegenseitig beeinflussen und kumulieren.

Der obligate Pflegeprozess [14] findet in der analogen und digital unterstützten Pflege lege artis nicht statt. Alle Pflegenden betrachten die SIS und die durch die Dokumentationsbeauftragen festgelegten Pflegemaßnahmen als Pflegeprozess. Weiter haben andere, extern beschäftigte Gesundheitsberufe, z. B. medizinische Fachangestellte, Pflegeaufgaben wie die Dekubitusversorgung übernommen. Dieser berufsgruppenübergreifenden Fragmentierung der Pflegearbeit folgt eine interpersonelle Fragmentierung der Pflegearbeit. Pflegehelfer*innen führen neben den Routineaufgaben auch unzulässigerweise Vorbehaltsaufgaben von Pflegefachpersonen durch, während diese durch Arbeiten in den Pflegestützpunkten der Wohnbereiche gebunden sind. Die Substitution durch Pflegende aus der ANÜ beurteilen die Pflegefachpersonen des Stammpersonals kritisch. *Hauptsächlich Arbeitnehmendenüberlassung, die wenig motiviert sind. Nur ihre Arbeit machen, ohne nachzudenken. Da kann nichts kommen (1. Gesprächsprotokoll PFP). *Die Konstellation aus doppelter Fragmentierung und Personalmangel verhindert erstens, dass die digitalen Möglichkeiten der Pflegebetten bewohnerorientiert genutzt werden, und zweitens, ein bewohner*innenorientierter Pflegeprozess zur Anwendung kommt. Unabdingbar ist es und beschrieben in Hasseler [6], dass für eine Optimierung der pflegerischen Versorgung und Erleichterung der Pflegearbeit die digitalen Optionen der Pflegebetten im Pflegeprozesses mitgedacht werden [5].

Weitere Forschungsergebnisse sind, dass vom Einrichtungsmanagement kein der Versorgungsrealität angepasstes Implementierungsmanagement durchgeführt wurde. Die Pflegefachpersonen wurden nicht befähigt, die digitalen Möglichkeiten der Pflegebetten zielgerichtet einzusetzen, um das pflegerische Outcome zu steigern. So könnten sie, wenn geschult, bei einem erhöhten Sturzrisiko die BEF aktivieren und so das Verletzungsrisiko für Bewohnende dadurch minimieren. Praktisch konnte keine Pflegeperson eines Pflegeteams die BEF aktivieren. Gemeinsam appellieren sie an die Bereichsleitung: *Bitte zeigst du uns das! *(Q: 00:20:49-4). Die Unkenntnis über die Möglichkeiten könnte somit zu einem mittelbaren Versorgungsrisiko für Bewohnenden führen. Pflegende suchen zudem nach alternativen Pflegehandlungen, wodurch der Versorgungsaufwand steigt. Dies führt zu einem Paradoxon: Der Unterstützungseffekt durch die Pflegebetten entfällt, und durch die steigende Arbeitsbelastung haben die Pflegenden keine Zeit, um die hilfreiche Technik zu erlernen, in der Praxis anzuwenden [15]. Die Datenanalysen zeigen, dass das ineffiziente Implementierungsmanagement das Paradoxon nicht auflöst und einer vorteilgenerierenden Inbetriebnahme der Pflegebetten entgegenwirkt. Eckstein und Burkardt (2021) beschreiben mit dem Begriff Stolpersteine hemmende Faktoren im Verlauf einer Implementierung [16]. Ein unzureichendes Engagement des Einrichtungsmanagements ist ein solcher Stolperstein.

### Schlussfolgerung

Es ist keine Illusion, dass digitale Assistenzsysteme (dAS) Pflegende in ihrer Arbeit aktiv unterstützen können. Mobile Monitoringsysteme beispielsweise von *complaint concept*® (Fehraltorf, Schweiz) unterstützen bereits bei der Sturz- und Dekubitusprävention [17]. Pflegefachpersonen sind technischen Neuerungen gegenüber offen eingestellt und nutzen sie [8]. Aber, je komplexer die Systeme sind, je tiefer sie in die Arbeitsstrukturen und vorhandene Systemen integriert werden müssen, umso höher steigen die Anforderungen an die dAS.

Im Fall der Pflegebetten sind die Anforderungen aus der Pflegepraxis an die Technik und Anwendungsfreundlichkeit nicht erfüllt worden. Die technischen Unzulänglichkeiten der Pflegebetten, der IT-Infrastruktur und die inadäquate Implementierung haben zum Verstreichen der Chance beigetragen, die Pflegebetten mit ihren Unterstützungs- und Entlastungspotenzial Teil des Pflegeprozesses und der praktischen Pflegearbeit zu werden. Der Einsatz einer neuen Technik führt wie in diesem Fall zu einer Intensivierung der Arbeit [18]. Beruflich Pflegende müssen die Defizite der Pflegebetten kompensieren, was u. a. zu Redundanzen in der Pflegearbeit führt. Die Ziele, die Pflegenden zu entlasten und Versorgungsqualität zu verbessern, sind nicht erreicht worden.

Diese Forschungsergebnisse zeigen, dass neue digitale Tools nicht per se wirken, sondern an pflegefachliche und technische Voraussetzungen geknüpft sind. Hoben et al. (2015) betonen in diesem Kontext die Notwendigkeit eines strukturierten Implementierungs- und Change-Managements [19].

## Ausblick

Offenkundiges Forschungsergebnis ist, dass die Pflegebetten nicht dem Bedarf der beruflich Pflegenden gerecht werden. Das Einrichtungsmanagement hat es versäumt, auf die problembehaftete Realität zu reagieren. Das beobachtete Phänomen der Problemresilienz auf Managementebene bekommt vor dem Hintergrund der Digitalisierung, verbundenen mit hohen (Fehl)Investitionskosten, eine neue Bedeutung und bedarf weiterer Forschung. Für eine Steigerung der Anwendbarkeit und der Akzeptanz neuer dAS in der Pflege gilt es zu erforschen, warum der gesetzlich vorgeschriebene Pflegeprozess von Entwickler*innen und Hersteller*innenseite nicht in der Entwicklungsphase mitgedacht wird. Ungeklärt in diesem Zusammenhang ist trotz hinlänglich bekannter Forderung aus der Forschung [20], warum beruflich Pflegende nicht bzw. nur marginal an Entwicklungs- und Verbesserungsprozessen beteiligt werden.

## Limitationen

Es bestanden keine Limitationen im Rahmen der Datenerhebung und Auswertung. Verzögerungen, die durch die COVID-19-Pandemie bedingt waren, konnten durch zeitliche Flexibilität kompensiert werden.

### Supplementary Information





