# Supplementary material for: Digital care beds in long-term care—Support, relief for caregivers or just illusion? An ethnographic explorative study
Source: Z Gerontol Geriatr. 2024 Jan 2;57(1):32–6. [Article in German] doi: 10.1007/s00391-023-02267-z (PMC10827873; doi:10.1007/s00391-023-02267-z)
Supplement: Supplementary file 1 [file 391_2023_2267_MOESM1_ESM.pdf]

Hinweise

Die Bildung der Kategorien/Codes erfolgt deduktiv, anhand bekannter Informationen aus der Fachliteratur, Vorwissen etc. und induktiv, aus Erkenntnissen der Interviews und Gesprächen. Eine Kategorie kann auch aus vorherigen Beobachtungen entstehen, wenn sie im Zusammenhang mit Interviewinhalten hat, z.B. Anwendungsfreundlichkeit. Folgende Anforderungen wurden an das Kategoriensystem gestellt:

- Name der Kategorie steht i.d.R. im direkten deduktiven bzw. induktiven Zusammenhang, bspw. Literatur/Interviewaussagen.
- Subkategorien resultieren aus einer notwendigen weiteren Differenzierung der Hauptkategorie zur Verfeinerung der Analyse. Subkategorien stellt aber keine Hierarchisierung untereinander dar.
- Die inhaltliche Beschreibung einer Kategorie ergibt sich aus dem induktiven wie deduktiven Kontext und werden als Codememo hinzugeführt.
- Anwendung der Kategorie: Eine Kategorie wird codiert, wenn der inhaltliche Aspekt auftritt. Z.B.:
    - Kategorie Ernährung: Wenn in der Gesprächssequenz bspw. Ernährungsprotokolle thematisiert werden.
- Optionale Anwendung einer Kategorie: Wenn Gesprächssequenzen auch wesentlich im Zusammenhang mit einer anderen Kategorie stehen. Z.B.:
    - Kategorie Dekubitus und Kategorie Pflege, mit Subkategorie fragmentierte Pflegearbeit. Dann kodieren, denn bspw. externe Berufsgruppen eine originäre Arbeit der Pflegefachpersonen durchführen.
- Wesentlich und das gilt für alle Kategorien/Codes ist, dass sie disjunkt sind. Was aber nicht bedeutet, dass die Aussage einer Sequenz nicht auf andere Kategorien zutreffen könnten.
- Mit der Kategorie Sonstiges können Aussagen codiert werden, die inhaltlich relevant sind, aber nicht direkt einer anderen (Sub)Kategorie zugeordnet werden können. Bspw. Organisationsstrukturen, -routinen.

Kodierleitfaden

Persönliche, situative Gedanken, Anmerkungen während der Analyse werden mit Memos in den Transkripten/Analysen festgehalten.

1. **Pflege:**
    - Pflegedokumentation, induktiver Code, kommt zur Anwendung bei direkten Aussagen zum Thema Dokumentation, egal ob analog oder digital.
    - Pflegeprozess: Alle Interviewaussagen, die direkt mit dem Pflegeprozess oder dem was in der Pflegeeinrichtung als solches angewandt wird oder zusammenhängt, bzw. dem zugeordnet werden könnte. Oder aber, Pflegenden erkennen nicht den Zusammenhang zu einen möglichen oder nötigen Pflegeprozess und dem Einsatz der digitalen Pflegebetten.
    - Pflegearbeit: induktiver Code bei allem, was im direkten Zusammenhang im Pflegearbeit beobachtet wird und nicht dem Pflegeprozess o.Ä. zugeordnet werden kann.
2. **Recht, Moral, Ethik**

Als induktiver Code kommt er zur Anwendung, wenn erkennbar ist, dass die Pflegenden die veränderte Situation im Zusammenhang mit den digitalen Pflegebetten reflektieren. Ihre subjektive Wahrnehmung oder Ansicht schildern. Die Binnenperspektive der Pflegeeinrichtung und Pflegenden ist relevant. Existiert ein ethischer Diskurs? Reflektieren die Pflegenden auch die digitalen Pflegebetten im Zusammenhang mit den Rechten der Bewohnenden und ihren Rechten als Arbeitnehmende. Verändern sich die Verantwortung bspw. durch ein Monitoring?

3. **Technik und Digitalisierung**

- Technikprobleme, induktiver Code aufgrund der ständigen technischen Probleme, die direkt oder indirekt mit den digitalen Pflegebetten in Verbindung stehen.
- Technikanwendung, induktiver Code. Da es nur um die Besonderheiten in der untersuchten Pflegeeinrichtung handelt.
- Achtung: Technikakzeptanz wird quantitativ ermittelt, durch ATI-Skala. Wird hier **nicht** kodiert.

4. **Kontextfaktoren**:

Sie haben maßgeblichen Einfluss darauf, ob und wie die digitalen Pflegebetten von den Pflegenden akzeptiert und v.a. in ihre Pflegearbeit integriert werden. Es gibt zahlreiche Kontextfaktoren.
Achtung: Kann eine Interview- Gesprächsaussage einem anderen Kontextfaktor ggf. einer anderen Kategorie zugeordnet werden? Die gelisteten Faktoren stellen Subkategorien dar.

- Implementierungsstrategie, induktiv gebildeter Code. Er wird codiert, wenn in Interviews eine Implementierungsstrategie des Management (übergeordneter Träger der Pflegeeinrichtung bzw. das Management der Pflegeeinrichtung selber) erkennbar ist. Oder seitens der Interviewten thematisiert wird, dass für sie eine Implementierungsstrategie gut, nicht ausreichend ist bzw. keine zu erkennen ist.
- Schulungen, deduktiver Code, da der Zusammenhang in der Literatur diskutiert wird und der Bettenhersteller eine initiale Schulung vor Ort durchgeführt hat. Dann eigenständiger induktiver Subcode geworden, da im Laufe des Forschungsprojektes die Relevanz des Themas Schulungen im Zusammenhang mit dem Implementierungsmanagement zugenommen hat.
- Bewohnende, induktiver Code. Aber weniger relevant geworden, da viele Bewohnende fit, überwiegend selbstständig und mobil sind. Zudem lehnen zum Teil Bewohnende oder bevollmächtigte Personen die möglichen digitalen Optionen ab, Stichwort: Gläserner Bewohnender, Persönlichkeitsrechte.
- Personalsituation, deduktiver Code aufgrund der allgemein bekannten prekären Personalsituation in der Pflege. Induktiv, da die Personalsituation vor Ort einen erheblichen Einfluss auf die Implementierung der digitalen Pflegebetten hat.

5. **Qualitätsindikatoren**:

Deduktiver Code. Dienen als Ergebnisindikatoren, sind zudem die häufigsten Probleme in der pflegerischen Versorgung in Pflegeeinrichtungen. Stürze, Dekubitus und Fehlernährung haben sofortige und/oder latente Auswirkungen auf die Gesundheit und Wohlbefinden der Bewohnenden. Die Prävention ist grundlegende Aufgabe der Pflegenden und kann durch digitale Assistenzsysteme unterstützt werden. Alle in den Protokollen enthaltenen Infos zu den Qualitätsindikatoren werden passend kodiert:

- Sturzgeschehen
- Dekubitus
- Ernährung

6. Ideen und Erwartungen

Induktiver Code zu den Hintergründen der Entscheidung, digitale Pflegebetten anzuschaffen. Zudem, welche Erwartungen waren/sind mit den Pflegebetten verbunden?

7. Sonstiges, allgemeines:

Wird kodiert, wenn eine wichtige Information nicht zu einer anderen Kategorie zugeordnet werden kann.

- Parallellaufende Erprobung eines anderen digitalen Assistenzsystems. Tauchen ähnliche/gleich Probleme auf?
- Beschrieben Routinen in der Pflegeeinrichtung, bzw. Arbeitszeiten, wie viele Pflegeplätze sind momentan belegt…
