# Supplementary material for: Digital care beds in long-term care—Support, relief for caregivers or just illusion? An ethnographic explorative study
Source: Z Gerontol Geriatr. 2024 Jan 2;57(1):32–6. [Article in German] doi: 10.1007/s00391-023-02267-z (PMC10827873; doi:10.1007/s00391-023-02267-z)
Supplement: Supplementary file 2 [file 391_2023_2267_MOESM2_ESM.pdf]

**(1) Einstieg**	**Erkenntnisinteresse**
Ich freue mich, dass Sie einem Interview zugestimmt haben. Ich möchte mich kurz selber vorstellen und den Ablauf des Interviews erklären, welche Rechte Sie haben. Das ganze Interview wird ca. 45-60 Minuten dauern.<br><br>Sie wissen ja, dass die Teilnahme freiwillig ist. Ich werde gleich beginnen, die Aufnahme zu starten. Würden Sie sich kurz vorstellen und bestätigen, dass Sie mit der Aufnahme des Interviews einverstanden sind und wissen, dass Sie jederzeit und ohne Folgen das Gespräch beenden können und das, wenn Sie es fordern, die Aufnahme gelöscht wird.<br><br>Nach der formalen Bestätigung stellen Sie sich bitte kurz vor, welche Funktion Sie hier ausüben, wie lange Sie im Beruf sind und wie lange Sie hier schon in dieser Einrichtung arbeiten, in welchen Stellenumfang.	Erste Kontaktaufnahme: Zuerst selbst vorstellen und erklären, dass alles freiwillig ist. jederzeit abgebrochen werden kann, Recht darauf, dass alle Daten gelöscht werden. Anonymität gewährleistet wird. Das Interview transkribiert wird und der Text dann quali. ausgewertet wird.

**(2) Personalsituation**	**Erkenntnisinteresse**
Intro: In den Vorgesprächen zum Projekt habe ich mit dem Management gesprochen. Ein Thema war u.a. die noch nicht zufriedenstellende Personalsituation.	
a) Hat sich die Personalsituation bei den Pflegefachpersonen in den letzten sechs Monaten verändert?<br>– Spielt Fluktuation eine Rolle?<br>b) Wie umfangreich muss das (Name der Pflegeeinrichtung) auf Arbeitnehmendenüberlassung (ANÜ) zurückgreifen?<br>c) Wie umfänglich werden die Pflegenden aus der ANÜ in die Nutzung der digitalen Pflegebetten eingewiesen?<br>d) Wie hoch ist die Bereitschaft, der Wille der Pflegenden mit den Betten zu arbeiten?<br>– Kommen Rückfragen, warum es nicht so gut läuft?<br>e) Hat die eher angespannte Personalsituation einen Einfluss auf die Einbindung der dig. Pflegebetten in die praktische Pflegearbeit?<br>f) Welche Gründe betrachten Sie noch als nachteilig?<br>– Hinweis: Haben bspw. Pflegefachpersonen zeitl. Ressourcen, die Handhabung des Bettes zu üben?<br>Fallen Ihnen sonst noch Zusammenhänge zwischen dem dig. Pflegebett und dem Personal ein?	Es ist wesentlich zu erfahren, wie „stabil" die Personaldecke ist. Seit Eröffnung im Frühjahr 2020 sind die Planstellen für das pfleg. Stammpersonal nicht besetzt. Wie schon von der PDL berichtet, wird viel mit ANÜ gearbeitet. Aber nur wenn das Stammpersonal „mannstark" besetzt ist, kann sich eine stabile Wissensbasis rund um das digitalen Pflegebett entwickeln, es in die Arbeitsroutinen integriert werden.

**(3) Pflegeprozess**	**Erkenntnisinteresse**
Intro: Der Pflegeprozess bildet die Grundlage professionellen Pflegens. Es gibt den wohl bekanntesten 6-stufigen Pflegeprozess nach Fichter und Meier. Daneben gibt es andere Varianten, bspw. der 4-stufige Pflegeprozess nach Yura/Walsh.	
a) Welcher Pflegeprozess kommt hier zur Anwendung und wie läuft der Pflegeprozess hier ab?<br>b) Welche Personen und Entscheidungsträger sind beteiligt, inwiefern darf die Pflegefachperson alleine entscheiden/handeln?<br>c) Wie, durch wen werden neu aufgetretene Pflegeprobleme erkannt und in den Pflegeprozess aufgenommen?<br>→ Wer entscheidet, wenn eine Pflegemaßnahme nicht mehr sinnvoll ist, sie abgesetzt werden soll?<br>→ Können Sie bitte den Prozess kurz am Beispiel eines auffällig veränderten Körpergewichtes (alternativ Dekubitus) beschreiben.<br>d) Welche Funktion im Pflegeprozess hat die Dokumentationsbeauftragte?<br>e) Wie unterstützt das digitale Pflegebett den Pflegeprozess im Moment?<br>f) Was sind die größten Hürden bei der Integration des Pflegebettes in die Pflegearbeit bzw. den Pflegeprozess?<br>g) Ist allen Pflegefachpersonen bewusst, dass wenn durch ein Assessment, bspw. dem Wiegen, Druckkurve… das durch das ermittelte Ergebnis eine Handlungsverantwortung entsteht, die bis auf die einzelnen Elemente des Pflegeprozesses zurückreicht?	Erkenntnisgewinn: Der obligate Pflegeprozess ist elementar für die strukturierte Pflegearbeit. Das dig. Pflegebett bietet Unterstützungsangebote (Waage, Bett-Exit-Funktion BEF). Werden diese Optionen zielführend für die pflegerische Versorgung genutzt? Weiter gilt es in Erfahrung zu bringen, auf welcher Entscheidungsebene als sinnvoll erachtete Pflegemaßnahmen genehmigt werden (müssen).

**(4) Digitales Pflegebett**	**Erkenntnisinteresse**
Intro: Das digitalen Pflegebetten ist ja nun seit kurzem/einigen Monaten im Einsatz. Es gibt ja noch paar technische Probleme. Aber so im Ganzen, wie ist der erste Eindruck, wie kommt es bei Ihnen und den Kolleginnen und Kollegen an?	
a) Kennen die Pflegefachpersonen des Stammpersonals bereits alle Funktionen des digitalen Pflegebettes?<br>b) Welche Funktionen nutzen die Pflegefachpersonen regelmäßig bei ihrer Pflegearbeit?<br>c) Gibt es Funktionen, Pflegemaßnahmen, die bspw. erst von Ihnen genehmigt werden müssen?<br>d) Ist es möglich, dass digitale Pflegebett für risikobehaftete Bewohnende (Dekubitus) in seiner jetzigen Form sinnvoll zu nutzen?<br>→ Wenn ja, was ist es im Besonderen?<br>→ Wenn nein, warum nicht?<br>e) Haben sich die Erwartungen, die mit dem digitalen Pflegebett entstanden sind erfüllt?<br>→ Welche besonders nicht?	Erkenntnisgewinn: Das Pflegebett bietet verschiedene technische Unterstützungsmöglichkeiten. Wie sieht es überhaupt mit der Nutzung aus, ist es möglich, diese Features zu nutzen? Nur wenn die Pflegenden einen Nutzen in der Anwendung der technischen Möglichkeiten sehen, werden diese genutzt.

f) Wie beurteilen Sie das Implementierungskonzept der digitalen Pflegebetten?	

**(5) Kleiners, paralleles digitales Forschungsprojekt eines Pflegeproduktеherstellers mit anderem Hintergrund in der Pflegeeinrichtung**	**Erkenntnisinteresse**
Intro: Das andere Forschungsprojekt weist kleine Überschneidungen mit dem Forschung zu den digitalen Pflegebetten auf. Vor allem in der Technikanbindung, Umsetzung und wie der Pflegeprozess darauf abgestimmt wird.	
a) Das Pilotprojekt zu (Name des Produktes) ist nun abgeschlossen. Wie beurteilen Sie im Vergleich zum Projekt mit den digitalen Pflegebetten die Unterstützung durch das Einrichtungsmanagement?<br>a. Unterstützung beim Changemanagement?<br>b. Technische Unterstützung durch IT-Abteilung, dem Einrichtungsmanagement?<br>b) Erkennen Sie Parallelen bei der Umsetzung bzw. Unterstützung durch das Einrichtungsmanagement<br>a. Problemfelder<br>b. Lösungsstrategien des Managements	Der Erkenntnisgewinn liegt primär auf die Implementierungsprozesse, in der Pflegeeinrichtung, im Zusammenhang mit dem Engagement und der Unterstützung durch das Einrichtungsmanagement.

**(6) Persönliche Einschätzung, Ausblick der interviewten Person**	**Erkenntnisinteresse**
Intro: In der qualitativen Forschung, vor allem bei Interviews sprechen wir von der sogenannten Zauberfrage. Antworten Sie einfach spontan, was Ihnen einfällt.	
a) Wenn Sie sich etwas wünschen könnten, Zeit, Geld, sonstige Ressourcen etc. spielen keine Rolle, was würden Sie sich wünschen, damit das Pflegebett optimal zum Wohle der Bewohnenden und Pflegefachpersonen genutzt werden kann?	Es gilt in Erfahrung zu bringen, welche weiteren Hindernisse oder auch Lösungsansätze aus der Perspektive der Pflegenden bestehen. Eine Idee, an die andere nicht gedacht haben…

(7) Abschluss/ Ausklang	Erkenntnisinteresse
Gibt es noch etwas von Ihrer Seite, dass Sie gerne noch im Zusammenhang mit dem Pflegebett erwähnen möchten, was Ihnen wichtig ist oder was bisher nicht zur Sprache gekommen ist?	Weitere Themen. Weitere Problemfelder, sonstige Erfahrungen. Gibt es Lösungsstrategien bei den Pflegenden?
Okay, damit wären wir am Ende des Interviews…	
Dann bedanke ich mich recht herzlich für das Interview und beende die Aufnahme.	

Postskript Interview:

- Ort:
- Zeitliche Einschätzung (bestand irgendwie Zeitdruck):
- Atmosphäre:
- Unterbrechungen:
- Antwortverhalten (nur gewünschte Antworten oder erkennbar frei, aus der Praxis):
- Sonstige besondere Vorkommnisse:
